# Salvage Intensity-Modulated Radiation Therapy (IMRT) for Locally Recurrent Nasopharyngeal Cancer after Definitive IMRT: A Novel Scenario of the Modern Era

**DOI:** 10.1038/srep32883

**Published:** 2016-09-12

**Authors:** Lin Kong, Lei Wang, Chunying Shen, Chaosu Hu, Lei Wang, Jiade J. Lu

**Affiliations:** 1 Department of Radiation Oncology, Fudan University Shanghai Cancer Center, Department of Oncology, Shanghai Medical College of Fudan University, China; 2 Department of Radiation Oncology, Shanghai Proton and Heavy Ion Center, Shanghai, China; 3 Department of Oncology, Second Hospital of Kashi, Xinjiang, China

## Abstract

Locally recurrent nasopharyngeal carcinoma (rNPC) after definitive IMRT occurs in 10% of all cases and represents a distinct clinical entity that has been selectively enriched by radio-resistant cancer cells. Therefore, we report of the outcomes of 77 patients who had repeat salvage-IMRT for rNPC after only a definitive course of IMRT. Various clinical outcomes were measured. Log-rank tests were used to detect differences in the survival outcomes between factor-defined subgroups. Multivariable analysis was performed using the Cox proportional hazard model. The median follow-up time was 25.7 months (range 3.0–75.7 months), measured from the time of recurrence. The median OS time and PFS time of the entire cohort was 37.0 and 20.5 months, respectively. Thirty-four patients (44.2%) died. Approximately 35% of these patients died from disease progression, but 53% were from treatment-induced severe adverse effects (SAEs) without evidence of disease progression. Higher T-classification of the recurrent tumor and the development of SAEs were found to be the only independent and significant adverse prognostic factors on multivariable analysis. These outcomes underscore the particularly virulent characteristics of rNPC after definitive IMRT. Concerning is the impact of re-irradiation toxicity on patient mortality.

Radiation therapy is the mainstay treatment modality for patients with non-metastatic nasopharyngeal cancer (NPC). The prevailing utilization of Intensity-modulated radiation therapy (IMRT) and dose localization through modern imaging techniques such as MRI has significantly improved the treatment outcome, especially the local control of NPC^1^. Nevertheless, local recurrence after IMRT remains a major mode of treatment failure. Approximately 10% of patients, especially those initially presenting with T3 or T4 lesions, continue to suffer from local recurrence^2^.

Few studies have reported on the outcomes of locally recurrent NPC re-irradiated using IMRT^3^,^4^,^5^. However, nearly all patients in these reports were treated in the pre-IMRT era and failed after their initial 2-dimentional conventional radiation therapy. Tumor volume coverage in these initial treatment courses could have been suboptimal, and the possibility of marginal miss of gross, but more often subclinical disease could have been more frequent as compared to IMRT. At the same time, dose distribution to normal nasopharyngeal mucosa surrounding the primary NPC lesion may have been uneven or poorly delineated.

Local recurrence of NPC after high-dose IMRT as initial treatment represents a unique condition and its management poses a new set of challenges to clinicians. Whereas recurrences in the previous eras could have been partially attributable to the technological shortcomings of treatment delivery, the recurrence of NPC that has been fully encompassed within the high-dose irradiation volumes represent a new biologic entity that could be more radio-resistant. Further, normal tissues such as mucosa, temporal lobes of the brain, cavernous sinus, and brain stem encompassed within the PTV of the recurrent irradiation has usually been irradiated intensely in the previous IMRT, especially for patients with locally advanced disease. As such, previous results of salvage IMRT for NPC after conventional radiotherapy may not be as readily applicable for NPC patients initially irradiated with IMRT. The purpose of this study is to address the outcomes, including toxicity profiles, of retreatment with IMRT in a group of NPC patients initially managed definitively with high-dose IMRT in a tertiary medical center.

## Methods and Materials

This observational study did not involve any human or animal experiments. All data collection and statistical analysis were conducted in accordance with the institutional Ethical Board of the Fudan University Shanghai Cancer Center. Informed consent was obtained on all subjects before their treatment. Informed consent for the study was waived based on the Ethical Board requirement for retrospective clinical studies.

### Patients and Pre-treatment Evaluation

Between May 2009 and May 2014, a total of 77 patients with locally or locoregionally recurrent NPC who failed initial IMRT with a curative intent were treated with salvage IMRT. Biopsy of the recurrent disease was performed in 48 (62.3%) patients, and the remaining 29 patients were diagnosed clinically with MRI and/or PET/CT. Total dose to the PTV of the primary gross disease was 66~70.4 Gy in the primary IMRT. Fifty-nine (76.6%) patients received chemotherapy as part of their initial treatment.

Pretreatment evaluation consisted of a complete history and physical examination, indirect nasopharyngoscopy, complete blood counts (CBC), and serum electrolyte levels, urinalysis, and MRI of the head and neck. Whole body PET/CT was optional, and CT of thorax/abdomen, ultrasound of the abdomen, and bone scan were required to rule out distant metastasis if PET/CT was not performed. All cases were re-staged based on the 7^th^ edition of the American Joint Cancer Committee staging classification.

Characteristics of the patients and their initial treatments are detailed in Table 1.

### Salvage Intensity-Modulated Radiation Therapy

All patients were immobilized with thermoplastic masks in supine position. CT simulation by use of 3-millimeter cuts from the vertex to 2 centimeters below the clavicular heads was performed. The last MRI of the head and neck performed after the diagnosis of recurrence were used for planning-CT fusion.

The gross tumor volume (GTV) of the recurrent disease in the base of skull and neck (GTV-P and GTV-N, respectively) included all recurrent lesions seen on imaging studies. The clinical target volumes (CTVs) of both GTV-P and GTV-N were designed to encompass microscopic disease by a margin expansion to 3~5 millimeters to both GTVs depends on the proximity to the critical OAR(s). Prophylactic irradiation to uninvolved neck lymph drainage areas was not provided. An additional 3-millimeter margin was added to the CTV to create the planning target volume (PTV).

OARs required for all patients were defined according to the following priority: brainstem, spinal cord, optic nerves/chiasm, temporal lobes, pituitary gland, eyes (including lens), temporomandibular joints, and parotid glands. Recovery from previous IMXT dose was set at 70%, based on the radiobiological conclusions of Nieder *et al*. regardless of the latent time between the two courses of radiation^6^. Dose limitation to the brain stem and spinal cord in re-IMRT were 40 Gy and 30 Gy to Dmax for all patients, respectively. The maximum dose allowed for other OARs were based on TD5/5 described by Emami with the consideration of previous irradiated dose with 70% recovery. For example, the dose constraint for re-irradiation for optic nerve/chiasm was set at 35 Gy if the initial radiation dose was 50 Gy (50 Gy (TD5/5) −30% X 50 Gy (initial dose to optic nerve/chiasm) = 35 Gy). Efforts were given to minimize the dose to any previously irradiated OARs.

Inverse treatment planning with a mono-isocentric technique with the Pinnacle treatment planning software system was used for all patients. Seventy-one patients received IMRT alone to a median dose of 66 Gy (range 46.2–70) prescribed to the PTV(s) at 2.0–2.1 Gy/day (5 days per week) including 3 patients received <60 Gy. Six patients received IMRT (50–56 Gy) plus brachytherapy boost. Treatment was by step-and-shoot IMRT with the use of 5–7 coplanar beams. All patients were examined weekly during radiation therapy.

### Chemotherapy

No standard chemotherapy protocol exists for the treatment of locally recurrent NPC in our institution. Cisplatin-based induction, concurrent, and/or adjuvant chemotherapy were administered to 55 patients with locally advanced recurrent NPC based on the discretion of the attending oncologists with the consideration of patients’ clinical needs and preference, including 12 patients (15.6%) received targeted agents (cetuximab or nimotuzumab). The utilization of various schedule of chemotherapy are detailed in Table 2.

### Follow-up

All patients were required to be followed-up every 3–4 months in the first 3 years, every 6 months for 2 additional years, and annually thereafter after the completion of their salvaging IMRT. Each follow-up visit included a complete history and physical examination, indirect nasopharyngoscopy, MRI of the head-and-neck regions, CBC, and serum electrolytes. CT of the thorax, ultrasound or CT of the abdomen, and/or PET/CT were optional and ordered based on clinical indication(s), so did pituitary function tests.

Treatment-induced adverse-effects were measured and recorded at each follow-up visit according to various versions of the Common Terminology Criteria for Adverse Events (CTC AE) at the time of measurement. Severe adverse effects (SAEs) were defined as Grade 3–5.

### Statistics

Overall survival time (OS) was measured from the diagnosis of local recurrence until confirmed death or the date of the last follow-up examination. The duration of local and regional progression-free and distant metastasis-free survival time (LPFS, RPFS, and DMFS), and progression-free survival time (PFS) were measured from the time of diagnosis of recurrent until documented locoregional or distant failure/progression. The rates of LPFS, RPFS, DMFS, PFS, and OS were estimated with the Kaplan-Meier method.

Prognosticators and predictive factors used for univariate and multivariate analyses included all significant factors previously published on re-irradiation using IMRT for locally recurrent NPC. Univariate Log-rank tests were used to detect differences in the survival outcomes between factor-defined subgroups. Multivariate analysis was performed using the Cox proportional hazard model. The level of significance was set at a two-tailed p value of <0.05.

## Results

The median interval between the completion of initial RT and the start of reirradiation for local recurrence was 27.9 (range 11.7–79.0) months for this cohort of patients. The median follow-up time was 25.7 months (range 3.0–75.7 months) measured from the time of diagnosis. Median time from the diagnosis of NPC recurrence to the initiation of re-IMRT was 2 months.

### Treatment Outcome and Cause of Death

**Figure 1:**
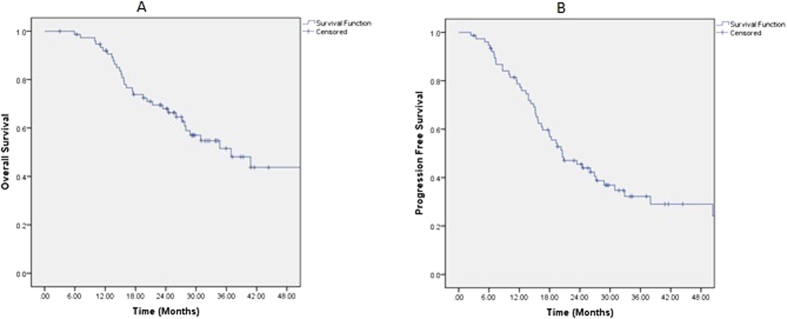
Overall survival (**A**) and Progression-Free Survival (**B**) after salvage IMRT for 77 nasopharyngeal carcinoma patients who failed their initial course of definitive IMRT.

At the time of this analysis, 34 patients (44.2%) had deceased. The median overall survival time and progression-free survival time of the entire cohort was 37.0 and 20.5 months, respectively (Fig. 1). The median time as well as the 1-, 2-, and 3-year OS, PFS, LPFS and RRFS rates were detailed in Table 3. Overall survival based on significant characteristics of disease and treatment outcome are detailed in Table 4. Approximately 35% patients died from disease progression, but 53% were from treatment-induced severe adverse effects (SAEs) without evidence of disease progression (Table 5) including 17 patients (50%) died of progressive mucosal necrosis which eventually caused massive hemorrhage.

### Toxicities

No patients experienced late SAE after their initial course of IMRT. However, late SAEs including mucosal necrosis, temporal lobe necrosis, and cranial neuropathy, trismus, and hearing deficit occurred in 50 (64.9%) of the 77 patients during their follow up (Table 6). Of note, the median time to developing mucosal necrosis was 4.6 months (range 0.6~63.9 months), and 67.7% of the mucosal necrosis occurred within 6 months after the completion of re-irradiation.

### Prognostic Factors

The value of all significant prognostic factors previously reported in the literature for locally recurrent nasopharyngeal cancer after re-irradiation using IMRT excluding tumor volume were assessed in both univariate and multivariate analyses. These potential prognosticators included age, gender, stage at initial diagnosis and recurrence, time to recurrence, use of chemotherapy, dose of re-IMRT to GTV, response to re-IMRT, and occurrence of SAE.

**Figure 2:**
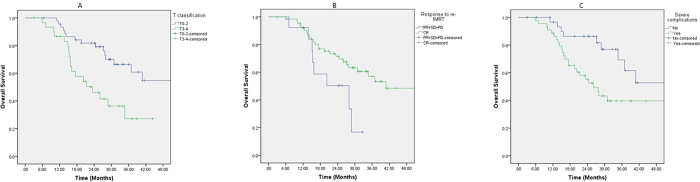
Overall survival after salvage IMRT for 77 nasopharyngeal carcinoma patients who failed their initial course of definitive IMRT based on: recurrent T-classification (**A**); response to re-irradiation with IMRT (**B**); and the occurrence of sever adverse events (**C**).

On univariate analyses, T-classification at recurrence was a significant prognosticator for OS, PFS, and DMFS. The occurrence of SAE that are potentially life threatening (i.e., mucosal necrosis, TLN, and CNP combined) was a significant prognostic factors for OS and PFS. And response to re-IMRT was significant for OS alone (Table 7, Fig. 1). The T-classification at disease recurrence and occurrence of SAEs were significant prognosticators for OS, and the occurrence of SAE was the only significant prognostic factors for PFS as well on multivariate analyses (Table 8, Fig. 2A,C).

## Discussion

IMRT remains the most important therapeutic modality for NPC patients who developed local recurrence after their initial radiotherapy. In this series, 77 NPC patients who failed their primary IMRT received a second course of high-dose IMRT for salvage at a tertiary comprehensive cancer center. With a median follow-up time of 25.7 months, the3-year OS, PFS, and LPFS were 51.5%, 32.3%, and 66.7%, respectively. Retreatment with IMRT seemed feasible and efficacious; nevertheless, approximately 50 (64.9%) of 77 of patients experienced severe late toxicity such as mucosal necrosis, cranial nerve neuropathy, and/or brain necrosis, hearing loss, trismus. Notably, 66.7% of the mucosal necrosis occurred within 6 months post salvaging IMRT. In addition, 35.3% of patients died from treatment failure after their second course of high-dose IMRT.

IMRT has been the most important modality for the salvaging of locally recurrent NPC in the recent years; however, only few studies from two institutions have reported long-term (i.e., ≥2 years) results. Collectively, outcomes were acceptable in the disease control of early recurrent-stage, but dismal results were observed in those patients who presented with a locally advanced condition at the diagnosis of recurrence. For example, the 5-year OS in patients with rT3 and rT4 disease were 32.3% and 37.9%, respectively, in a large series of 239 patients^4^. In addition, the 5-year OS of patients with more advanced nodal recurrence (i.e., N2 or N3 disease) was less than 15%. Results from the earlier data presented from the same institute as well as those from another retrospective study also suggested outcomes in the same range, and the reported 2- or 3-year OS were 34–54% in patients with rT3 or rT4 disease in three other series^3^,^5^.

Nearly all patients included in the above-mentioned studies were initially treated with conventional 2D-RT, whereas all patients reported in our series failed their initial definitive IMRT. Despite the similarities in the radiation techniques, doses, and other patient- or treatment-related characteristics involved in the salvage treatment of our patients, our results revealed that the 3-year overall survival rate in patients with rT3 or rT4 disease were merely 27.2%, substantially lower than those reported previously who failed their initial, conventional therapy. In addition, at the time of the last follow up, local recurrence occurred in ~10% of the patients after IMRT reirradiation in patients who failed 2D-RT; however, the 3-year LPFS was merely 66.7% in our series. These phenomena suggested that NPC locally failed after high-dose IMRT have more dismal outcome as compared to those who failed 2D-RT. IMRT is the standard radiotherapy technique for definitive treatment of NPC. Local recurrence after initial IMRT, although occurs less frequently as compared to 2D-RT, clearly represents the current pattern of the NPC management.

It is reasonable to postulate that locally recurrent NPC after high-dose IMRT has a different and more virulent biological behavior and represents a distinct clinical condition. With the advances in imaging technology and radiation technology, improved tumor volume coverage ensured that both gross and subclinical tumor volumes are sufficiently encompassed in the CTV. In addition, most local failures are within or overlap with the high-dose CTV after IMRT^1^. Clearly, dosimetric insufficiency, i.e., marginal miss during the initial course of treatment, does not form a good explanation of local failure after IMRT in NPC. Inherent resistance to irradiation could be one of the main reasons for treatment failure after IMRT for NPC.

A detailed analysis for potential mechanisms of radio-resistance in recurrent NPC is out of the scope of this clinical report. However, a number of studies have indicated that nasopharyngeal cancer stem cells (CSCs) or stem cell like cells may be induced by previous radiotherapy therapy and be responsible for the resistance to further salvage treatment^7^,^8^,^9^,^10^. In addition, NPC stem cell like cells may also be responsible for resistance to chemotherapy^11^. It is not surprising that all reported data have indicated that chemotherapy was not a significantly prognostic factor in the re-irradiation of NPC local recurrence using IMRT.

Prognostic factors for patients with locally recurrent NPC re-treated with IMRT has not been consistent in published literature due to, at least in part, the inhomogeneous nature of patients treated by different centers. In the initial results reported by Hua *et al*., only rT-classification and recurrent tumor volume were found to be significant in predicting OS after re-irradiation with IMRT^3^. However, in their more recently published series with more sample size, age, rN-classification, recurrent stage, tumor volume, and mean fractional dose were found to be significant in predicting the overall survival. Age and tumor volume were the only significant prognosticator for local recurrence-free survival (LRFS) and distant metastatic-free survival (DMFS), respectively^4^. Additional publications from the same group with longer follow-up confirmed certain but not all factors^12^. However, in an earlier report by Qiu *et al*. the original T-classification at initial diagnosis and time to recurrence were significant in the prediction of OS, disease-free survival (DFS), and LRFS^5^. As this is the first study on the outcome after salvage IMRT after definitive IMRT for NPC, we tried to included all potential indicators used in the previous publications in our multivariate analyses for the purpose of completeness except for the recurrent tumor volume. Our results demonstrated that although the r-T-classification, response to re-irradiation, and occurrence of SAEs were significant prognostic factors for OS, PFS, and/or DMFS in univariate analyses, the occurrence of SAEs and high r-T-classification were the only two poor prognostic indicators for OS in multivariate analyses. In addition, the occurrence of SAEs was the only significant prognosticator for PFS.

SAEs from re-irradiation are the most important cause of treatment failure. After conventional RT, the covered volume of the surrounding mucosa is usually larger, and the dose distribution is inhomogeneous. On the other hand, although IMRT can spare more mucosa that is farther away from the lesion, higher and uniform doses are usually delivered in the encompassed normal tissues. In a small series of 31 conventionally treated patients re-irradiated with IMRT with or without radiosurgery, although 70% of patients developed late toxicities, most cases were mild. Grade 3 toxicities (cranial neuropathy, ototoxicity, brain necrosis, and soft-tissue fibrosis) were observed in 7% and 25% of cases at 6 and 12 months respectively. And brachytherapy or radiosurgery used in the primary radiation therapy course indicated significantly higher probability of Grade 3 toxicities after re-irradiation^13^. SAEs such as mucosal necrosis, CNP, or brain necrosis which could be life threatening reported in other salvage IMRT series was up to 40%. Furthermore, severe late adverse effects and local progression were the cause of 69.2% and 10.8% of patients’ death, respectively^4^. Similarly, approximately 40%, 9%, and 26% of patients developed grade 3~5 mucosal necrosis, brain necrosis, and severe cranial neuropathy in our series, respectively. However, it is important to note that although mucosal necrosis might develop at any time, 67% of mucosal necrosis in our series occurred within 6 months after the complete of re-irradiation. The earlier development of mucosal necrosis and deaths it caused was different from the patterns previously reported and was due to, at least in part, the radiation technique (IMRT) used in initial treatment. As mentioned above, although the PTV of IMRT usually encompass only the mucosa areas close to the primary lesion in the post nasal space, higher and more uniform dose was usually delivered to these areas. Furthermore, our multivariate analyses indicated that SAE was the only significant prognosticators for both OS and PFS. This finding is not surprising as we consider the local failures after suboptimal treatment delivery versus a more radio-resistant condition differ from each other. Early onset of SAEs may have severely masked the frequency of local progression in NPC patients who failed IMRT and were salvaged with repeated IMRT.

As far as we know, this is the first report on the outcome of salvage IMRT in locally recurrent NPC patients who failed their primary IMRT course. Unfortunately, comparison between this cohort of patients directly to those IMRT-salvaged patients failed after initial 2D-RT in the same period was not possible. IMRT technology has been widely implemented as the standard technology for definitive treatment of non-metastatic NPC for more than a decade, thus patients treated with conventional RT in the 2D era would be either cured or failed long time ago. Another potential pitfall of the current study is that the the median follow-up time of 26 months was relatively short, thus we only reported our 3-year outcome. Nevertheless, a recently published large series from our institution on IMRT for NPC indicated that most of the NPC recurrences, whether local or distant, occur within the first 2–3 years after the completion of IMRT^14^. Therefore, we consider our 3-year results are able to approximate the long-term outcome. In addition, nearly 70% of the most common late effect in our series, i.e., soft tissue necrosis, occurs within the first 6 months after the completion of salvaging IMRT.

The poor prognosis in NPC patients who failed their primary IMRT clearly indicated that a safer and more efficacious therapeutic modality is needed. As the cause of deaths are mostly due to treatment-induced SAEs, more physically accurate and biologically effective local treatment technique is needed to improve the outcome. Although brachytherapy and stereotactic ablative body radiotherapy (SABR) can be used for small lesions, their utilization is limited by the extent of the disease^15^,^16^.

Particle therapy such as proton or carbon-ion radiation therapy (CIRT), may represent a more favorable option, as it provides distinct physical characteristics that include a sharp lateral penumbra, very low energy deposition within the entry path prior to the Bragg peak formed by the steep dose deposition, and a sharp dose fall-off after the Bragg peak, thus possessing a dose delivery with a finite range. Sparing of normal surrounding tissues is crucial in radiation therapy of head and neck area especially patients who have completed a previous course of high-dose radiation. These dose-deposition characteristics lead to a significant reduction in the integral dose delivered and overall OAR volume affected. This is especially important for the patients presented in our current study, given the high mortality rate from SAEs from repeat IMRT.

A number of studies have reported superior dose distributions using particle therapy for primary or recurrent NPC with acceptable clinical outcomes and improved dosimetry^17^,^18^ when compared to photon-based treatments. In addition to its superior physical properties, CIRT has high LET, and its relative biological effectiveness (RBE) is significantly higher than those of photon and proton radiation. The value of RBE is 3~5 for carbon ion depend on the tissue type and end point of study. It has been suggested that more damage from high LET radiation is in the form of direct DNA double strand breaks, which is more difficult to repair^19^. In addition, CIRT is more effective in targeting CSCs as compared to photon radiation based on *in vitro* results^20^,^21^. As such, improved clinical results could be expected after high-LET radiation such as CIRT especially for photon-resistant cancer cells.

The use of CIRT in the setting of heavily irradiated sites has been reported for adenoid cystic carcinoma, chordoma/chondrosarcoma with favorable outcome^22^,^23^. An early study on neon and helium ion beams for locally recurrent NPC also indicated the feasibility and potential effectiveness, although modern imaging technique was not used for diagnosis and tumor volume delineation^24^. Two phase I/II trials with the aims of defining the maximal tolerated dose (MTD) of CIRT and the efficacy at such dose, with or without chemotherapy, in our institute^25^,^26^. The results of these studies will provide initial data in the use of heavy particle in the management of locally recurrent NPC.

## Conclusion

Local recurrence in NPC after high-dose IMRT may represent a distinct clinical condition with more suboptimal outcomes after salvage treatment with a second course of IMRT. Re-irradiation with IMRT provided 3-year LPFS, DFS, and OS rates of 66.7%, 32.3%, and 51.5%, respectively. Treatment-induced SAE occurred in 65% of patients and is the most important contributor to mortality in this group of patients. Newer treatment strategies and modalities are needed to improve the treatment outcome including late toxicity in NPC patients who recurred locally after IMRT.

## Additional Information

**How to cite this article**: Kong, L. *et al*. Salvage Intensity-Modulated Radiation Therapy (IMRT) for Locally Recurrent Nasopharyngeal Cancer after Definitive IMRT: A Novel Scenario of the Modern Era. *Sci. Rep.* **6**, 32883; doi: 10.1038/srep32883 (2016).

## Figures and Tables

**Table 1:** Characteristics of the 77 patients with locally recurrent NPC and their initial treatment.

Characteristics	No. (%)
Gender	
Male	57 (70.4%)
Female	20 (26.0%)
Age at local recurrence	
Median (range)	46 yr (30~77)
≤50 years	46 (59.7%)
>50 years	31 (40.3%)
Initial treatment	
IMRT alone	8 (10.4%)
IMRT with chemotherapy	69 (89.6%)
Initial radiation technique (IMRT)	100%
Stage at initial diagnosis	
I and II	28 (36.4%)
III and IVa/b	49 (63.6%)
Time to recurrence	
Median (range)	25.5 mos (5.8–83.4)
Stage at recurrence	
I and II	46 (59.7%)
III and IVa/b	31 (40.3%)
Recurrent T/N-classification	
r-T0	8
r-T1	29 (33.8%)
r-T2	9 (11.7%)
r-T3	20 (24.7%)
r-T4	11 (14.3%)
r-N+	20 (26.0%)
Neck lymph node only	12 (15.7%)

**Table 2:** Schedule of chemotherapy in 55 patients with locally advanced recurrent NPC.

Chemotherapy Schedule		No. (%)
Induction	Induction only	40 (51.9%)
	+concurrent	6 (7.8%)
	+adjuvant	2 (2.6%)
	+both	1 (1.3%)
Concurrent	Concurrent only	4 (5.2%)
	+adjuvant	1 (1.3%)
Adjuvant	Adjuvant only	1 (1.3%)
None		22 (28.6%)

**Table 3:** Treatment outcomes (survival) of the 77 locally recurrent NPC patients.

	Median (months)	1-year	2-year	3-year
OS	37.0 (95% CI:24.4–49.5)	92.0%	68.0%	51.5%
PFS	20.5 (95% CI:14.5–26.6)	78.7%	45.5%	32.3%
LPFS	59.3 (95% CI:37.1–81.4)	89.1%	76.9%	66.7%
RRFS	Not reached	95.9%	86.7%	81.4%
DMFS	Not reached	90.5%	79.9%	77.4%

**Table 4:** Overall survival based on significant characteristics of disease and treatment outcome.

Characteristics		Median survival	1-year	2-year	3-year	P value
rT	rT0–2	62.3 (29.0–95.6)	95.6%	81.8%	66.4%	
	rT3–4	23.4 (13.5–33.3)	86.7%	46.1%	27.2%	**0**.**004**
Response of re-RT	CR	40.8 (22.5–59.1)	91.9%	71.5%	57.1%	
	PR + SD + PD	28.0 (12.0–43.9)	92.3%	50.3%	16.8%	**0**.**038**
SAEs	—	62.3 (62.3–62.3)	96.7%	86.3%	69.3%	
	+NP necrosis, TLN, CNP	26. 1 (20.2–32.0)	88.6%	55.3%	39.7%	**0**.**034**
Mucosal necrosis	—	62.3 (62.3–62.3)	97.7%	88.1%	68.8%	**0**.**000**
	+Mucosal necrosis	20.3 (13.4–27.2)	83.9%	40.0%	27.7%	

**Table 5:** Cause of deaths in 34 deceased patients after salvage IMRT.

Cause	Number	Percentage (%)
Disease progression	12	35.3
Other disease	1	2.9
SAEs	18	52.9
Unknown	3	8.8
Total	34	100

**Table 6:** Grade 3–5 toxicities of the 77 patients with local recurrent NPC.

SAE type	Number (%)
Mucosal necrosis	31 (40.3%)
TLN	7 (9.1%)
CNP	20 (26.0%)
Trismus	18 (23.4%)
Hearing loss	4 (5.2%)

**Table 7:** Univariate analysis of potential prognostic factors.

Factors		OS	PFS	LPFS	RPFS	DMFS
Gender	male vs. female	0.668	0.117	0.330	0.211	0.433
Age	≤50 years vs. >50 years	0.463	0.536	0.352	0.670	0.665
Initial stage	I/II vs. III/IV	0.612	0.926	0.535	0.324	0.453
Time to recurrence after initial IMRT	≤2 years vs. >2 years	0.888	0.274	0.947	0.507	0.530
r-T classification	T0–2 vs.T3–4	***0***.***004***	***0***.***057***	0.305	0.106	***0***.***059***
r-N category	N0 vs. N1–2	0.913	0.295	0.224	0.784	0.318
Chemotherapy	Yes vs. No	0.698	0.739	0.439	0.886	0.191
GTV dose	≤66 Gy vs. >66 Gy or IMRT + Brachy	0.699	0.516	0.593	0.656	0.184
Response to re-IMRT	CR vs. PR + SD + PD	***0***.***038***	0.140	0.652	0.199	0.921
SAE (Mucosal necrosis, TLN, CNP)	Yes vs. No	***0***.***034***	**0**.**000**	0.579	0.895	0.405

SAE – severe adverse effects; TLN – temporal lobe necrosis; CNP – cranial nerve palsy.

**Table 8:** Multivariate analysis of potential prognostic factors for OS and PFS.

		OS			PFS		
Factors		HR	95% CI	P value	HR	95% CI	P value
Gender	male vs. female	0.819	0.356–1.885	0.639	0.615	0.289–1.310	0.208
age	≤50 years vs. >50 years	1.014	0.644–1.597	0.952	0.919	0.646–1.306	0.637
Initial stage	I/II vs. III/IV	1.311	0.611–2.812	0.487	0.636	0.329–1.228	0.178
Time to recurrence after initial IMRT	≤2 years vs. >2 years	0.686	0.302–1.561	0.369	1.140	0.599–2.169	0.689
r-T category	T0–2 vs.T3–4	1.478	1.021–2.141	***0***.***038***	1.253	0.918–1.711	0.155
r-N category	N0 vs. N1–2	1.266	0.469–3.420	0.641	0.740	0.305–1.794	0.505
Chemo	Yes vs. No	0.742	0.315–1.751	0.496	0.699	0.341–1.435	0.330
GTV dose	≤66 Gy vs. >66 Gy or IMRT + Brachy	0.901	0.398–2.041	0.803	0.769	0.404–1.464	0.425
Response to re-IMRT	CR vs. PR + SD + PD	0.590	0.213–1.634	0.310	0.734	0.312–1.722	0.477
SAEs (Mucosal necrosis, TLN, or CNP)	Yes vs. No	1.324	1.003–1.748	***0***.***047***	1.405	1.118–1.767	***0***.***004***
